# The Impact of Hospital Size on National Trends and Outcomes Following Open Esophagectomy

**DOI:** 10.3390/medicina55100669

**Published:** 2019-10-03

**Authors:** Sameer A. Hirji, Rohan M. Shah, Adam Fields, Vwaire Orhurhu, Nizar Bhulani, Abby White, Gita N. Mody, Scott J. Swanson

**Affiliations:** 1 Division of Thoracic Surgery, Department of Surgery, Brigham and Women’s Hospital, Harvard Medical School, Boston, MA 02115, USA; SHIRJI@partners.org (S.A.H.); Rshah0@partners.org (R.M.S.); awhite12@bwh.harvard.edu (A.W.); 2 T. H. Chan Harvard School of Public Health, Boston, MA 02115, USA; ACFields@partners.org (A.F.); vwo569@mail.harvard.edu (V.O.); nizar.bhulani@gmail.com (N.B.); 3 Division of Cardiothoracic Surgery, University of North Carolina, Chapel Hill, NC 27599-7065, USA; gita_mody@med.unc.edu

**Keywords:** open esophagectomy, hospital size, esophageal cancer

## Abstract

*Background and Objectives*: Previous studies have demonstrated superior patient outcomes for thoracic oncology patients treated at high-volume surgery centers compared to low-volume centers. However, the specific role of overall hospital size in open esophagectomy morbidity and mortality remains unclear. *Materials and Methods:* Patients aged >18 years who underwent open esophagectomy for primary malignant neoplasia of the esophagus between 2002 and 2014 were identified using the National Inpatient Sample. Minimally invasive procedures were excluded. Discharges were stratified by hospital size (large, medium, and small) and analyzed using trend and multivariable regression analyses. *Results*: Over a 13-year period, a total of 69,840 open esophagectomy procedures were performed nationally. While the proportion of total esophagectomies performed did not vary by hospital size, in-hospital mortality trends decreased for all hospitals (large (7.2% to 3.7%), medium (12.8% vs. 4.9%), and small (12.8% vs. 4.9%)), although this was only significant for large hospitals (*P* < 0.01). After controlling for patient demographics, comorbidities, admission, and hospital-level factors, hospital length of stay (LOS), total inflation-adjusted costs, in-hospital mortality, and complications (cardiac, respiratory, vascular, and bleeding) did not vary by hospital size (all *P* > 0.05). *Conclusions*: After risk adjustment, patient morbidity and in-hospital mortality appear to be comparable across all institutions, including small hospitals. While there appears to be an increased push for referring patients to large hospitals, our findings suggest that there may be other factors (such as surgeon type, hospital volume, or board status) that are more likely to impact the results; these need to be further explored in the current era of episode-based care.

## 1. Introduction

Significant advances have been made over the last half-century in the management of patients with esophageal cancer. In particular, the availability of efficacious neoadjuvant and adjuvant therapies, innovations in thoracoscopic techniques, and the emergence of sophisticated imaging platforms have together spurred an era of innovation and creativity in the management of complex patients, who often present with advanced disease [1,2]. Despite tangible improvements in overall disease morbidity and mortality, the five-year overall survival rate is still relatively high, ranging from 19% to as high as 43% in certain scenarios, especially in patients who undergo open esophagectomy [3]. Thus, there is a constant need to identify various patient-related, surgeon-related, and hospital-related factors that can contribute towards improved outcomes after esophagectomy, as these patients are often deemed high-risk surgical candidates for mortality and readmissions [4].

There is a growing body of literature demonstrating the impact of operative volume and/or hospital volume on overall patient outcomes [5,6,7,8]. In particular, these studies have shown a strong correlation between esophagectomy volume and patient outcomes, especially in patients treated at high-volume surgery centers [5,6,7,8]. While these findings are encouraging, volume alone has been shown to be an inadequate proxy of quality assessment after esophagectomy [9]. To address these inadequacies, the Society of Thoracic Surgeons (STS) recently utilized the General Thoracic Surgery Database (GTSD) to develop composite quality measure scores for measuring the performance of hospitals performing esophagectomies. Even though the utility and reliability of GTSD have been validated previously [10,11], the composite scores were not generalizable (as they only included voluntary institutions participating in the database) and were also prone to selection bias (as they excluded almost 60% of institutions performing fewer than five esophagectomies annually, i.e., the low-volume centers) [10,11].

To our knowledge, there is no study that has specifically examined the impact of overall hospital size on national trends and outcomes (morbidity and mortality) specific to open esophagectomy. We hypothesized that hospital size may play a significant role in terms of outcomes, in addition to known factors such as surgeon and hospital volume. To explore this hypothesis further and to utilize a more reliable, nationally representative sample of hospitals performing esophagectomy in the United States, we used the National Inpatient Sample, an administrative database, to examine the impact of hospital size on national trends and outcomes. This research may provide useful benchmarking data to guide clinical decision-making and provide a framework to improve quality and transparency at a national level. Thus, in this study, we sought to (1) describe contemporary national trends in open esophagectomy surgery volume and mortality; (2) examine the impact of hospital size on patient outcomes; and (3) develop a predictive model for in-hospital mortality following open esophagectomy.

## 2. Materials and Methods

### 2.1. Data Source

Data were obtained from the National (Nationwide) Inpatient Sample (NIS), the largest publicly available, all-payer administrative claims-based database, approximating a 20% sample of all discharges from about 1000 nonfederal participating hospitals from 45 U.S. states. The NIS is sponsored by the Agency for Healthcare Research and Quality (AHRQ) as part of the Healthcare Cost and Utilization Project (HCUP), and contains de-identified patient discharge information on demographics, comorbidities, in-hospital diagnoses and procedures, admission costs, and hospital-level characteristics for each patient hospitalization. These data are sampled from de-identified hospitals and weighted by region and year, using probability weights to obtain national estimates. This study was considered exempt from institutional review board approval as the NIS contains de-identified patient information.

### 2.2. Study Population

We identified hospitalizations in patients aged ≥18 years who underwent open esophagectomy surgery between 2002 and 2014. To isolate this cohort, we used the International Classification of Diseases, Ninth Revision, Clinical Modification (ICD-9-CM) procedure codes highlighted in Table S1. We first isolated all patients who carried a primary diagnosis of primary esophageal cancer. We then excluded patients who did not undergo an esophagectomy. We also excluded patients who underwent a minimally invasive esophagectomy for multiple reasons. First, a specific procedure code for minimally invasive esophagectomy does not exist. Furthermore, we found inconsistent ICD-9 coding for laparoscopic procedures over time. Given the de-identified nature of hospitals in the NIS, we were unable to control for these coding practices at the hospital level. Finally, the presence of substantial variability in technique and volume across different hospitals cannot be adequately adjusted for given the de-identified nature of the NIS and the need to utilize probability weights to generate national estimates.

### 2.3. Study Outcomes

Our primary outcomes of interest were national trends in procedures and all-cause, in-hospital mortality. Secondary outcomes of interest included acute myocardial infarction (MI), stroke, major bleed, acute kidney injury (AKI), hospital length of stay (LOS), inpatient cost, and discharge disposition following surgery. A list of ICD-9-CM codes used to define in-hospital complications is also included in Table S1.

### 2.4. Statistical Analysis

Utilizing survey analysis procedures, we utilized the sample of discharges to generate weighted national estimates and variances that accounted for clustering of outcomes within hospitals and sampling variation across strata (region and year) as recommended by AHRQ. A Cochran‒Armitage test for trends was conducted to determine significant differences in open esophagectomy volume and mortality over time. Open esophagectomy surgeries were then stratified by hospital size (small, medium, or large), as defined by the NIS [12]. Briefly, size categories were based on the number of hospital beds, and were specific to the hospital’s region (Northeast, Midwest, South, West) and teaching status (teaching vs. nonteaching), as the NIS uses different cutoff points for rural, urban nonteaching, and urban teaching hospitals (see Table S2). Patient demographic information, clinically relevant diagnoses, surgical and in-hospital outcomes, hospital LOS and costs, and discharge disposition were extracted and compared by size. AHRQ comorbidities were utilized both as single categories and in the Charlson comorbidity index, to serve indirectly as an indicator for frailty. In addition to size, the hospitals’ location and teaching status were also examined.

Normally distributed continuous variables were expressed as a mean with standard deviation and compared using one-way analysis of variance (ANOVA) tests. Categorical variables were presented as number and percentages and compared using χ^2^ tests. We then adjusted the outcomes for small hospitals using multivariable logistic and linear regression to control for patient demographics, comorbidities, admission, and hospital-level factors. Independent predictors for in-hospital mortality were determined by including all pre-operative variables, which were then included in a backward selection, parsimonious multivariable logistic regression model with *p* < 0.05 as the threshold for inclusion. The final model contained pre-operative variables that met the threshold for inclusion along with hospital size, given the latter’s significance for this project. Since a medium hospital size was an independent risk factor in our predictive model, we performed a subgroup analysis to further explore predictors of in-hospital mortality within this hospital size category. This second logistic regression model was performed similarly to the previous model. Missing data were rare (<1% for all variables). A *P*-value of <0.05 was the threshold criterion for statistical significance for all tests and models. Analysis was conducted using STATA Version 13.1 (StataCorp LP, College Station, TX, USA).

## 3. Results

### 3.1. National Trends in Procedures and In-Hospital Mortality

**Figure 1:**
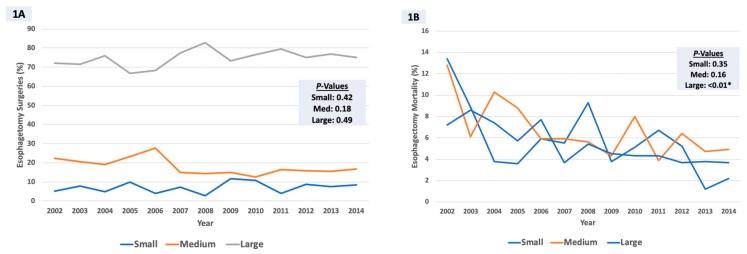
(**A**) Surgical volume and (**B**) in-hospital mortality temporal trends for open esophagectomy procedures by hospital size.

Over a 13-year period (2002–2014), 69,840 open esophagectomy procedures were performed. Large hospitals accounted for the majority of the procedures (*n* = 52, 344; 75%) followed by medium-sized (*n* = 12, 451; 17.8%) and small hospitals (*n* = 5, 045; 7.2%). The total number of operations increased annually from 4956 in 2002 to 5460 in 2014, though the increase was not statistically significant (*P* = 0.97 for trend). The proportion of total esophagectomies performed did not vary by hospital size (all: *P* > 0.05; Figure 1A). In-hospital mortality trends decreased for all hospitals (large (7.2% vs. 3.7%), medium (12.8% vs. 4.9%), and small (12.8% vs. 4.9%)), although this was only significant for large hospitals (*P* < 0.01; Figure 1B).

### 3.2. Patient Demographic and Clinical Characteristics

Patients undergoing esophagectomy surgery at large hospitals were significantly younger (63.5 vs. 63.7 and 64 years, *P* < 0.01) than in medium and small hospitals (Table 1). However, gender, race, household income, and patient-payer mix were similar between the three groups (all *P* > 0.05). Likewise, the three groups had similar patient comorbidities, except that large hospitals had a higher proportion of patients with depression and a higher Charlson comorbidity index (both *P* < 0.05). The majority of the surgeries in large hospitals were done electively (90.4%), compared to 89.2% in small and 87.7% in medium-sized hospitals (*P* = 0.04). On the other hand, medium-sized hospitals had a higher proportion of patients with prior myocardial infarction (16.4%) compared to large (13.7%) and small hospitals (14.8%; *P* < 0.01). There was also a statistically significant difference in terms of hospital ownership (government vs. private), teaching status, and geographic region/location between the three hospital groups (all *P* < 0.05).

### 3.3. In-Hospital Outcomes and Disposition Tendencies

The incidence of major in-hospital cardiac, vascular, bleeding, and respiratory-related outcomes did not significantly vary between small, medium, and large hospitals (Table 2). Likewise, disposition tendencies following open esophagectomy were not statistically significant (*P* = 0.48), although there was a higher tendency among small hospitals to transfer their patients to either short-term hospitals (2% vs. 1.2% and 0.9%) or skilled nursing facility/rehabs (16.6% vs. 15.3% and 15.6%) compared to medium-sized and large hospitals, respectively.

In terms of in-hospital mortality, the unadjusted mortality was similar between small and large hospitals (5.2% for both), but significantly lower than medium-sized hospitals (6.9%; *P* = 0.01). Notably, small hospitals also had a significantly unadjusted shorter hospital LOS (15.9 vs. 16.8 and 16.5 days) and unadjusted decreased total inflation-adjusted admission healthcare costs ($73,413 vs. $78,635 and $74,752; all *P* < 0.01) compared to medium and large hospitals, respectively. However, after controlling for patient demographics, comorbidities, admission, and hospital-level factors, hospital LOS, total inflation-adjusted costs, and in-hospital mortality did not vary by hospital size (all *P* > 0.05; see Table S3).

### 3.4. Predictive Model

The results of our multivariable logistic regression model for predicting in-hospital mortality following open esophagectomy are summarized in Table 3. Importantly, a medium-sized hospital (odds ratio (OR) 1.48; reference: small hospital), coagulopathy (OR 2.99), liver disease (OR 2.37), fluid and electrolyte disorders (OR 2.19), congestive heart failure (OR 1.85), renal failure (OR 1.45), age (OR 1.04/year), recent weight loss (1.46), and nonelective status of surgery (OR 1.83) were independent predictors of in-hospital mortality. Large hospital size was not an independent predictor of mortality (*P* = 0.55; reference: small hospital).

In our subgroup analysis, we further examined the independent factors associated with increased in-hospital mortality among medium-sized hospitals. These were insurance status (Medicaid and self-insured compared to Medicare patients), congestive heart failure, liver disease, nonelective admission, weekend admission, weight loss, Charlson comorbidity index, and age (all *P* < 0.01; see Table S4).

## 4. Discussion

Our study takes an in-depth look at the impact of hospital size on national trends and in-hospital outcomes following open esophagectomy in the United States. Using a national representative sample, this study led to several important findings. First, we demonstrated that while the total number of operations increased annually from 2002 to 2014, the proportion of total esophagectomies performed did not vary by hospital size. However, in-hospital mortality trends decreased for all hospitals (large (~2-fold), medium (~3-fold), and small (~3-fold)), although this was only significant for large hospitals (*P* < 0.01). Secondly, although large hospitals accounted for more than 75% of these surgeries, the resource allocation of sicker patients undergoing open esophagectomy at larger hospitals resulted in comparable risk-adjusted patient morbidity and in-hospital mortality to small and medium-sized hospitals. Finally, only medium hospital size (as opposed to large) was associated with an increased risk of in-hospital mortality (almost 50% higher) when compared to small hospitals, after adjusting for various patient and hospital risk factors. These estimates provide useful data for benchmarking hospital performance in the context of open esophagectomy, and emphasize the need for closer scrutiny at hospitals (especially medium-sized ones) that may be underperforming relative to their peers.

The existing literature has shown a strong association between the volume of esophagectomies and patient outcomes [13,14]. For esophagectomies, data from a Canadian database of 6985 patients showed that high-volume centers (>20 cases per year) had 64% decreased odds of in-hospital mortality and 38% decreased LOS [13]. Furthermore, a recent study by Fuchs et al. utilized the NIS from 1998 to 2011 and found that the overall perioperative mortality rate after esophagectomy was 7.7%, but it was higher in low-volume centers (11.4%) versus high-volume centers (4.01%) [5]. In a multivariable regression, high hospital volume had a protective effect on mortality (OR 0.54; 95% CI 0.45–0.65) [5]. Unfortunately, hospital volume alone has been shown to be an inadequate predictor of outcomes [9]. This is because, in addition to hospital volume, there are other factors that are equally important and tend to play a pivotal role in determining overall patient outcomes. These include surgeon experience and training level, which is a key factor associated with improved outcomes. Other factors include the utilization of multidisciplinary teams such as medical oncology, radiation oncology, nursing staff, intensive care unit staff, and physical therapy. Hospital size, in our opinion, is also an important factor because most hospitals (especially large and medium-sized hospitals) are often affiliated with major cancer centers, and tend to play a crucial role in patient referrals and providing outpatient ancillary cancer care. Recent studies have also shown an association between hospital size and patient-reported outcomes, including overall patient satisfaction [15,16]. Thus, there is a greater incentive for hospitals to find ways to improve outcomes and the quality of care as they accommodate more referred patients.

More recently, the STS GTSD task force proposed a composite performance measure to evaluate the quality of care in patients undergoing esophagectomy for esophageal cancer. The composite score was derived from two-risk adjusted outcomes (i.e., mortality and major complications) using participants from 2012 to 2014, and compared against the NIS as a benchmark (which included non-STS participants) [17,18]. Overall operative mortality was 3.1% and the rate of major complications was 33.1%. The advantage of the STS GTSD database is that it uses prospectively collected clinical data from voluntary participants, and provides more granular, patient-level risk adjustment, which is a limitation of administrative databases such as the NIS [17,18]. The downside of using it for benchmarking performance is the lack of generalizability or inclusiveness [10]. For instance, while the composite rating had good reliability for programs performing an average of five or more procedures annually, more than 60% of participants were ineligible for a star rating as they did not have a sufficient patient volume. Even though the overall estimates of in-hospital mortality were relatively higher in our cohort (5.2% for small and large hospitals and 6.9% in medium-sized hospitals), they are more representative of real-world practice patterns. We did not look specifically at hospital volume, but rather at hospital size. Nonetheless, our findings may be useful for patient counseling, surgical planning, resource allocation, and risk stratification.

Understanding the financial implications of hospital size is essential is important in the context of Medicare reimbursements because patient outcomes (especially mortality and readmissions) are closely scrutinized and penalized [19,20]. Thus, there may be an impetus for ‘bad practices’ in order to avoid financial penalties. For instance, small hospitals may transfer their high-risk patients to large hospitals, SNF/rehabs, or short-term hospitals. On the other hand, medium-sized or large hospitals could refuse transfers of sicker patients because theoretically they could negatively affect their overall mortality and readmission rates. Given the nature of the database, granular information on interhospital transfers and/or reasons for transfer was not available. In our study, we found a higher tendency among small hospitals to transfer their patients to either short-term hospitals (almost 2-fold) or SNF/rehabs (16.6% vs. 15.3% and 15.6%) compared to medium-sized and large hospitals, respectively. Likewise, we found that large hospitals saw sicker patients with a higher comorbidity burden (i.e., a higher Charlson comorbidity index). Despite these high-risk patients, it was reassuring to see the decreasing in-hospital mortality trends in large hospitals. While the exact reasons are unknown, a couple of factors could have influenced this trend, including the fact that large hospitals could have more experienced surgeons, the availability of qualified multi-disciplinary teams, and well-established post-discharge care coordination facilities for complex patients, all of which could have contributed towards improved patient outcomes. Importantly, however, while patients should always be referred to a specialist, they should also factor in hospital-level factors, especially in terms of the availability of multidisciplinary personnel and infrastructure capacity.

Additionally, while existing concerted efforts in establishing surgical benchmarks are commendable, there is significant variability between hospitals, which is often a function of location, the patient mix (demographics), skilled staffing, teaching versus nonteaching, and surgeon-related factors (volume, training). Thus, existing metrics should utilize a combination of these factors in order to provide a better distinction between high- and low-performing hospitals. Further research, however, is warranted to explore the utility of this approach.

The results of our study should be interpreted in light of both its strengths and limitations. The NIS is derived from hospital claims data, without access to individual medical records, and is subject to the shortcomings of administrative datasets. Inconsistent coding practices among institutions may have resulted in over- or underestimations of patient comorbidities and hospital outcomes, although HCUP quality control measures are in place to minimize these discrepancies. Sampling practices of the NIS also vary from year to year, as hospitals enter and leave the sampling frame, resulting in possible over- or undersampling by the study design. Despite our best efforts to use a validated coding scheme, residual confounding and misclassification may exist. The *n* of the mortality analysis for small and medium-sized hospitals was lower compared to large hospitals, which could also be the reason for the *p*-values being nonsignificant. Our results may also only be applicable to the U.S. hospital system, where no systematic specialization and centralization is present. We also did not examine the impact of hospital volume, as this relationship has been previously shown. Thus, our findings should be interpreted with caution.

Furthermore, we were unable to examine long-term outcomes beyond a single admission, which limited our ability to assess trends and the effect of hospital size on readmissions and aggregate costs after the index hospitalization. Due to the nature of our database, frailty could not be measured directly. The NIS also does not contain details on patient presentation, cancer pathology, surgeon experience, decisions, and surgical procedures, which could be important.

## 5. Conclusions

In this large, 13-year, observational cohort study of open esophagectomy procedures, we found that, after risk adjustment, patient morbidity and in-hospital mortality appear to be comparable across all institutions, including small hospitals. While there appears to be an increased push for referring patients to large hospitals, our findings suggest that hospital size does not matter. Rather, there may be other factors (such as surgeon type, hospital or operator volume, or board status) that are more likely to impact the results and need to be further explored in the current era of episode-based care.

**CENTRAL PICTURE**

Visual summary of key findings and implications of this study.

**CENTRAL MESSAGE**

After risk adjustment, patient morbidity and in-hospital mortality after open esophagectomy appear to be comparable across all institutions, and independent of the impact of hospital size.

**PERSPECTIVE STATEMENT**

Resource allocation of sicker patients undergoing open esophagectomy at larger hospitals results in comparable risk-adjusted patient morbidity and in-hospital mortality to small and medium-sized hospitals. Our findings suggest that there may be other factors (such as surgeon type or board status) that are more likely to impact patient outcomes in the current era of episode-based care.

## Figures and Tables

**Table 1:** Patient demographics, comorbidities and admission characteristics, stratified by hospital size.

Cohort Characteristics	Hospital Size (*n* = Number of Open Esophagectomies)			
Variable	Small (*n* = 5045)	Medium (*n* = 12,451)	Large (*n* = 52,344)	*P*-Value
Demographics				
Age	64.0 (10.8)	63.7 (10.6)	63.5 (10.6)	<0.01 *
Female	836 (16.6)	2161 (17.4)	9406 (18.0)	0.49
Race				0.62
White	3470 (85.5)	8860 (84.4)	37,629 (86.7)	
Black	156 (3.8)	498 (4.7)	1810 (4.2)	
Hispanic	253 (6.2)	669 (6.4)	2118 (4.9)	
Asian or Pacific Islander	85 (2.1)	236 (2.2)	712 (1.6)	
Payer				0.24
Medicare	2131 (42.3)	5808 (46.7)	24,252 (46.4)	
Medicaid	329 (6.5)	740 (6.0)	3154 (6.0)	
Private	2307 (45.8)	5393 (43.4)	22,851 (43.7)	
Self-Paying	163 (3.2)	157 (1.3)	766 (1.5)	
***Median Household Income Quartile per Zip Code***				***0.43***
1	975 (19.7)	2485 (20.5)	9977 (19.5)	
2	1322 (26.7)	2889 (23.9)	12,517 (24.5)	
3	1409 (28.5)	3274 (27.0)	13,426 (26.3)	
4	1241 (25.1)	3467 (28.6)	15,194 (29.7)	
Comorbidities				
Alcohol abuse	241 (4.8)	510 (4.1)	2144 (4.2)	0.59
Deficiency anemias	963 (19.2)	2365 (19.2)	8148 (15.8)	0.06
Chronic blood loss anemias	98 (2.0)	268 (2.2)	890 (1.7)	0.38
Congestive heart failure	289 (5.8)	742 (6.0)	2699 (5.2)	0.34
Chronic pulmonary disease	1223 (24.4)	2840 (23.0)	11,006 (21.3)	0.12
Coagulopathy	368 (7.3)	723 (5.9)	2921 (5.7)	0.33
Depression	237 (4.7)	575 (4.6)	3168 (6.1)	<0.01 *
Diabetes, uncomplicated	973 (19.4)	2037 (16.5)	8398 (16.3)	0.08
Diabetes with chronic complications	40 (0.8)	203 (1.6)	972 (1.9)	0.07
Hypertension	2494 (49.7)	5782 (46.9)	24,667 (47.8)	0.37
Liver disease	132 (2.7)	289 (2.3)	1208 (2.3)	0.85
Fluid and electrolyte disorders	1603 (32.0)	3392 (27.5)	15,218 (29.5)	0.24
Obesity	329 (6.6)	924 (7.5)	3896 (7.5)	0.59
Peripheral vascular disorders	262 (5.2)	423 (3.4)	1954 (3.8)	0.1
Renal failure	145 (2.9)	476 (3.9)	1776 (3.4)	0.37
Rheumatoid arthritis/Collagen vascular diseases	64 (1.3)	116 (0.9)	668 (1.3)	0.35
Weight loss	792 (15.8)	2204 (17.9)	8444 (16.4)	0.38
Charlson comorbidity index	4.0 (2.7)	4.3 (2.8)	4.3 (2.8)	<0.01 *
Prior radiation	632 (12.5)	1182 (9.5)	5105 (9.8)	0.20
Atrial fibrillation	1115 (22.1)	2748 (22.1)	11,175 (21.4)	0.71
Smoking	1589 (31.5)	3989 (32.0)	15,695 (30.0)	0.23
COPD	96 (1.9)	275 (2.2)	850 (1.6)	0.16
Prior MI	745 (14.8)	2040 (16.4)	7186 (13.7)	0.01 *
Prior TIA/Stroke	715 (14.2)	1732 (13.9)	7875 (15.1)	0.38
Admission Characteristics				
Admission on weekend	82 (1.7)	453 (3.6)	1594 (3.0)	0.26
Elective admission	4484 (89.2)	10,902 (87.7)	47,241 (90.4)	0.04 *
Hospital Factors				
Control/ownership of hospital				<0.01 *
Government, nonfederal	254 (5.0)	199 (1.6)	2900 (5.5)	
Private, not-for-profit	1067 (21.2)	3064 (24.6)	14,538 (27.8)	
Private, investor-owned	328 (6.5)	680 (5.5)	2508 (3.6)	
Location/teaching status of hospital				<0.01 *
Rural	38 (0.7)	79 (0.6)	2052 (3.9)	
Urban nonteaching	481 (9.5)	2528 (20.3)	9699 (18.5)	
Urban teaching	4526 (89.7)	9843 (79.1)	40,593 (77.6)	
Region of hospital				0.01 *
Northeast	740 (14.7)	2083 (16.7)	13,162 (25.2)	
Midwest	1420 (28.1)	2634 (21.2)	12,808 (24.5)	
South	1879 (37.2)	4331 (34.8)	17,189 (32.8)	
West	1007 (20.0)	3402 (27.3)	9184 (17.6)	

Abbreviations as follows: COPD—Chronic obstructive pulmonary disorder, MI—Myocardial infarction, TIA—Transient ischemic attack. Continuous variables are presented as mean (SD) unless otherwise noted as median (IQR); categorical variables are summarized as *n* (%). * A *P*-value ≤ 0.05 was considered statistically significant.

**Table 2:** In-hospital outcomes and disposition tendencies, stratified by hospital size.

Patient Outcomes	Size of Hospital (*n* = Number of Open Esophagectomies)			
Variable	Small (*n* = 5045)	Medium (*n* = 12,451)	Large (*n* = 52,344)	*P*-Value
In-Hospital Outcomes				
Acute myocardial infarction	90 (1.8)	217 (1.7)	731 (1.4)	0.32
Acute kidney injury	415 (8.2)	903 (7.3)	4125 (7.9)	0.54
Cardiac arrest	46 (0.9)	198 (1.6)	577 (1.1)	0.09
Major bleed	226 (4.5)	626 (5.0)	2754 (5.3)	0.53
Vascular complications	210 (4.2)	552 (4.4)	2254 (4.3)	0.94
Stroke	27 (0.5)	111 (0.9)	305 (0.6)	0.19
Aspiration	280 (5.5)	850 (6.8)	2919 (5.6)	0.17
Pulmonary insufficiency	657 (13.0)	1444 (11.6)	6902 (13.2)	0.33
Post-operative cardiac complications	412 (8.2)	1071 (8.6)	4192 (8.0)	0.66
Pneumonia	795 (15.8)	1757 (14.1)	7123 (13.6)	0.18
Reintubation	738 (14.6)	1585 (12.7)	6259 (12.0)	0.10
Reoperation for bleeding	0 (0.0)	5 (0.04)	21 (0.04)	0.82
Death	260 (5.2)	852 (6.9)	2738 (5.2)	0.01 *
LOS (days)	15.9 (12.4)	16.8 (14.4)	16.5 (15.2)	<0.01 *
Cost (USD, inflation adjusted)	73, 413 (67,706)	78,635 (81,076)	74,752 (74,304)	<0.01 *
Disposition (not including death)				0.48
Routine	1904 (37.7)	4683 (37.7)	19,129 (36.6)	
Transfer to short-term hospital	99 (2.0)	143 (1.2)	486 (0.9)	
Transfer to SNF, ICF, rehab	838 (16.6)	1904 (15.3)	8146 (15.6)	
Home health care	1944 (38.5)	4815 (38.8)	21,747 (41.6)	

Abbreviations as follows: SNF—Skilled nursing facility, ICF—Intermediate care facility, LOS—Length of stay. Continuous variables are presented as mean (SD) unless otherwise noted as median (IQR); categorical variables are summarized as *n* (%). *A *P*-value ≤ 0.05 was considered statistically significant.

**Table 3:** Multivariable logistic regression for independent predictors of in-hospital mortality after open esophagectomy.

Variable	Odds Ratio	95% Confidence Interval		*P*-Value
Small Hospital Size	(ref)			
Medium Hospital Size	1.48	1.04	2.10	0.03 *
Large Hospital Size	1.11	0.81	1.51	0.55
Coagulopathy	2.99	2.42	3.69	<0.01 *
Liver Disease	2.37	1.68	3.33	<0.01 *
Fluid and Electrolyte Disorders	2.19	1.86	2.59	<0.01 *
Congestive Heart Failure	1.85	1.44	2.39	<0.01 *
Nonelective Status	1.83	1.47	2.28	<0.01 *
Weight Loss	1.46	1.22	1.76	<0.01 *
Renal Failure	1.45	1.07	1.97	0.02 *
Age	1.04	1.03	1.05	<0.01 *

* A *P*-value ≤ 0.05 was considered statistically significant.

## Supplementary Materials

The following are available online at https://www.mdpi.com/1010-660X/55/10/669/s1, Figure S1: Study CONSORT diagram, Table S1: Relevant ICD-9 diagnoses and procedure codes, Table S2: Hospital size categories (number of beds), by region based on NIS sample, Table S3: Adjusted in-hospital outcomes in small bed-size hospitals using multivariable regression, Table S4: Multivariable logistic regression for independent predictors of in-hospital mortality in Medium-size hospitals.
