# Supplementary material for: The Impact of Hospital Size on National Trends and Outcomes Following Open Esophagectomy
Source: Medicina (Kaunas). 2019 Oct 3;55(10):669. doi: 10.3390/medicina55100669 (PMC6843198; doi:10.3390/medicina55100669)
Supplement: Supplementary file 1 [file medicina-55-00669-s001.pdf]

medicina

MDPI

Supplementary Materials

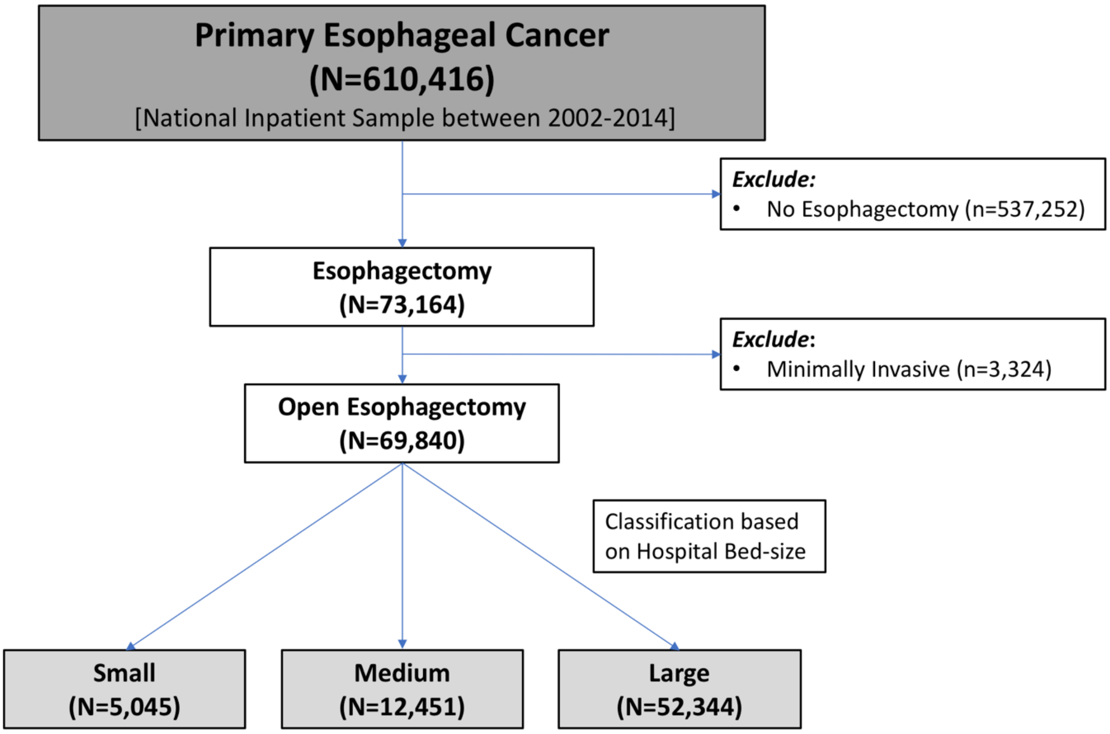

Figure S1. Study CONSORT diagram.

Table S1. Relevant ICD-9 Diagnoses and Procedure Codes.

**Diagnosis/ Procedure**	**ICD-9 Code(s)**
Acute Kidney Injury	584.XX
Acute Myocardial Infarction	410.XX, 4111
Aspiration	5304, 9973
Atrial Fibrillation	42731
Cardiac Arrest	4275
COPD	49121, 49000, 490, 4910, 4911, 4912, 49120, 49121, 49122, 4918, 4919
Esophageal Cancer	1500, 1501, 1502, 1503, 1504, 1505, 1508, 1509, 1510, 2301
Esophagectomy	424, 4240, 4241, 4242, 4399, 4252, 425
Laparoscopic	5421
Major Bleed	430.XX, 432.XX, 336.XX, 36243, 36281, 36361, 36362, 36372, 36441, 37742, 37923, 37923, 4560, 45620, 5307, 53082, 53100, 53101, 53121, 53140, 53141, 53160, 53161, 53200, 53201, 53220, 53221, 53240, 53241, 53260, 53261, 53300, 53301, 53320, 53321, 53340, 53341, 53360, 53361, 53400, 53401, 53420, 53421, 53440, 53441, 53460, 53461, 5693, 5780, 5781, 5789, 56881, 59970, 59971, 7191.X, 7847, 7848, 459.XX, 99811, 99812"
Pneumonia	480.XX, 481.XX, 482.XX, 483.XX, 484.XX, 485.XX, 486.XX, 487.XX

Post-operative Cardiac Complications	9971, 4230, 4233, 370
Prior Myocardial Infarction	3051, 412.XX
Prior Radiation	V1530
Prior TIA/ Stroke	V1254, 414.XX, 438.XX
Pulmonary Insufficiency	5184, 5185, 5188
Reintubation	9604
Reoperation for Bleeding	3941, 3949, 3988
Smoking	3051, V1582
Stroke	99702, 99700, 99701, 9970, 36231, 36812, 7814, 43311, 431.XX, 434.XX, 435.XX, 3446
Surgical Complications	9981, 9982, 9983
Vascular Complications	900.XX, 901.XX, 902.XX, 903.XX, 904.XX, 9982, 9992, 9977, 4470, 86804, 4410, 44100, 44101, 44102, 44103, 4411, 4413, 4415, 4416, 41410, 41419, 41412, 44322, 4440, 44401, 444109, 4441, 4442, 44421, 44422, 4448, 44481, 44489, 4449

*Alcohol Abuse, Deficiency Anemia, Rheumatoid Arthritis/ Collagen Vascular Diseases, Chronic Blood Loss Anemia, Congestive Heart Failure, Chronic Pulmonary Disease, Coagulopathy, Depression, Diabetes (uncomplicated & with chronic complications), Hypertension, Liver Disease, Fluid and Electrolyte Disorders, Obesity, Peripheral Vascular Disorders, Renal Failure, and Weight Loss were defined using the AHRQ comorbidity definitions included in the NIS database.

Table S2. Hospital Size Categories (in Number of Beds), by Region based on NIS sample.

Location and Teaching Status	Hospital Bed Size		
	Small	Medium	Large
**NORTHEAST**			
**Rural**	1 - 49	50 - 99	100+
**Urban, non-teaching**	1 - 124	125 - 199	200+
**Urban, teaching**	1 - 249	250 - 424	425+
**MIDWEST**			
**Rural**	1 - 29	30 - 49	50+
**Urban, non-teaching**	1 - 74	75 - 174	175+
**Urban, teaching**	1 - 249	250 - 374	375+

SOUTH			
**Rural**	1 - 39	40 - 74	75+
**Urban, non-teaching**	1 - 99	100 - 199	200+
**Urban, teaching**	1 - 249	250 - 449	450+
**WEST**			
**Rural**	1 - 24	25 - 44	45+
**Urban, non-teaching**	1 - 99	100 - 174	175+
**Urban, teaching**	1 - 199	200 - 324	325+

Table S3. Adjusted In-Hospital Outcomes in Small bed-size hospitals using Multivariable Regression

Outcome	OR	95% CI		P-value
Acute Myocardial Infarction	1.04	0.52	2.09	0.91
Acute Kidney Injury	1.00	0.74	1.35	0.99
Cardiac Arrest	0.76	0.34	1.72	0.51
Major Bleeding Event	0.87	0.63	1.19	0.38
Vascular Complications	0.96	0.65	1.4	0.80
Stroke	0.76	0.22	2.71	0.68
Surgical complications	0.80	0.52	1.23	0.31
Aspiration	1.09	0.65	1.81	0.75
Pulmonary insufficiency	1.00	0.73	1.37	0.98
Post-operative cardiac complications	1.01	0.69	1.47	0.96
Pneumonia	1.05	0.84	1.31	0.70
Reintubation	1.17	0.89	1.54	0.27
Reoperation for bleeding	1.13	0.76	1.43	0.74
Mortality	0.93	0.64	1.37	0.73
Linear Regressions				
**Outcome**	**Beta Coefficient**	**95% CI**		**P-value**
ln (LOS)	0.01	-0.03	0.05	0.54
ln (Cost)	-0.03	-0.11	0.05	0.45

-Binary outcomes adjusted using logistic regression. Represented as Odds Ratio (OR) with 95% Confidence Interval (CI). Model adjusted for patient demographics, comorbidities, admission and hospital-level factors.

- Length of stay (LOS) and cost adjusted using linear regression.

**P*-value ≤ 0.05 was considered statistically significant.

Table S4. Multivariable logistic regression for independent predictors of in-hospital mortality in Medium-size hospitals.

Variable	Odds Ratio	95% Confidence Interval		P-value
Medicare status	(ref)			
Self-Insured	2.00	1.53	2.61	<0.01*
Medicaid status	1.38	1.17	1.62	<0.01*
Congestive Heart Failure	1.67	1.46	1.91	<0.01*
Liver Disease	1.52	1.25	1.85	<0.01*
Non-Elective Admission	1.5	1.34	1.67	<0.01*
Weight Loss	1.32	1.2	1.46	<0.01*
Weekend Admission	1.18	1.07	1.31	<0.01*
Charlson Comorbidity Index	1.11	1.09	1.13	<0.01*
Age	1.02	1.01	1.02	<0.01*

**P*-value ≤ 0.05 was considered statistically significant.
